# The implementation of salt reduction strategies should be sped up in Africa: a shout from Morocco

**DOI:** 10.11604/pamj.2020.37.340.27388

**Published:** 2020-12-14

**Authors:** Jean Jacques Noubiap

**Affiliations:** 1 Centre for Heart Rhythm Disorders, University of Adelaide and Royal Adelaide Hospital, Adelaide, Australia

**Keywords:** Salt reduction, hypertension, cardiovascular disease, Africa

## Editorial

Cardiovascular disease (CVD) remains the leading cause of mortality and a major contributor to disability globally [1]. High dietary sodium is one of the most important risk factors for CVD. It is associated with hypertension and increased risk of stroke and heart disease [2]. According to the Global Burden of Disease study, in 2019, high dietary sodium accounted for an estimated 1.7 million cardiovascular deaths worldwide, representing ~10% of the overall cardiovascular mortality [2]. In view of the huge burden of high dietary sodium, salt reduction has been identified by the World Health Organization (WHO) as one the most efficient and cost-effective measures that can improve population health outcomes [3]. The WHO recommends that adults consume less than 2 g of sodium per day which corresponds to less than 5 g (just under a teaspoon) of salt per day [3]. The Global Burden of Diseases Nutrition and Chronic Diseases Expert Group (NutriCoDE) estimated the global mean sodium intake at 3.95 g per day in 2010, almost twice the recommended maximum level of intake [4]. The WHO projected that nearly 2.5 million deaths could be prevented each year if global salt consumption were reduced to the recommended level [3].

In May 2013, the 66^th^ World Health Assembly endorsed the WHO Global Action Plan for the Prevention and Control of Noncommunicable diseases 2013-2020. This initiative adopted a global target of a 30% reduction in mean population salt intake by 2025, as one of nine targets to achieve a 25% relative reduction in premature mortality from non-communicable diseases by 2025 [5]. Key strategies to reduce salt intake include (i) creating a legislation framework to mandate food labelling and compliance to recommended levels of dietary salt in processed foods; (ii) establishing appropriate fiscal policies to ensure the availability and affordability of low-salt products; (iii) raising population awareness on the need to reduce salt consumption; (iv) monitoring of population salt intake, sources of salt in the diet and consumer knowledge, attitudes and behaviours relating to salt to inform policy decisions [3].

A systematic review of progress towards the global target of salt reduction defined by the WHO showed that 75 countries had developed a national salt reduction strategy in 2014 [6], compared with 32 countries in 2010 [7]. Across these 75 countries, the most common components of their strategies were consumer education (n = 71 countries), industry engagement to reformulate products (n = 61), interventions in public institutions (n = 54), legislation to support salt reduction actions (n = 33), and front-of-pack labelling schemes (n = 31) [6]. As a result, reduction in population salt consumption was reported in 12 countries, reduced salt content in 19 countries and improved consumer knowledge, attitudes and behaviours relating to salt in 6 countries [6]. Unfortunately, at the time, only South Africa and Morocco had developed a salt reduction strategy among African countries [6].

In their study published in this volume of the Pan African Medical Journal, Bouhamida and colleagues examined the knowledge about the national strategy of salt reduction, and the implementation of the recommendation of salt reduction in bread among bakers in Morocco [8]. A total of 432 bakeries from all administrative regions of Morocco were surveyed using a questionnaire. They found that most bakers (73%) were not aware of the recommendations on the progressive reduction of the salt content in bread. Worse still, none of the bakers was informed about the process of gradual reduction of the level of salt in bread, and of course, none of them was implementing it. Furthermore, up to 60% of bakeries were not compliant to the national recommendation of 10 g of salt per kg of flour. However, the large majority of bakers (90%) were willing to implement the recommendations on the progressive reduction of the salt content in bread [8]. These findings in bakeries in Morocco, one of the first country to develop a salt reduction strategy in Africa, are suggestive of a worse situation in other African countries.

Interventions targeting bakeries are vital for a salt reduction strategy. Indeed, bread is one the most consumed foods and a major source of dietary sodium in most populations worldwide [9-14]. In South Africa, bread accounts for up to 40% of sodium intake [10]. In Morocco, bread is the most consumed food, with an average daily consumption of half a kilogram per individual [11]. Furthermore, with a mean of 8 to 9 g of salt per bread, most Moroccan adults reach the recommended maximum level of sodium intake only with bread consumption [12]. Other studies in Nigeria and Mozambique have shown that the sodium content of bread from bakeries and traditional markets was far above the recommended daily allowance [13,14]. Therefore, bakeries should be a central target in salt reduction strategies. Beyond a legislation to mandate compliance to recommended levels of salt in bread, bakers should be educated and trained on the process of gradually reducing the salt content in bread, and the implementation of this intervention should be rigorously monitored.

South Africa has achieved significant success in sodium regulation. In 2013, South Africa put in place a sodium legislation setting mandatory restrictions of sodium content across a wide range of commonly consumed processed foods [15]. The regulation was enacted to reduce sodium levels in the target foods in two waves; the first came into force in June 2016 and the second, with lower sodium targets, in June 2019 [15,16]. A study assessed the sodium levels of packaged foods in South Africa during the one-year period preceding the mandatory implementation date of this legislation [17]. It showed that two-thirds of foods covered by the sodium legislation already met the sodium target during early stages of policy implementation. However, almost three-quarters of breads, the major source of non-discretionary sodium intake in South Africa, did not meet legislated limits, highlighting the need to give a special attention to bakeries in the salt reduction process [17]. In fact, the decision by the South African government to enforce mandatory regulation on food industries was mainly informed by a modelling study that suggested that a 0.85 g reduction of daily sodium intake per individual could prevent 7400 cardiovascular deaths; 6400 of which would be due to lowering the sodium content of bread alone [18].

Considering the increasing burden of hypertension and CVD in African populations [1,2,19-21], governments should strongly commit to the implementation of a salt reduction strategy. This is particularly crucial in sub-Saharan Africa, as black populations are more sensitive to salt, i.e., more susceptible to sodium-related increase in blood pressure and to its adverse impact on health outcomes [22,23]. The achievements of South Africa in implementing a mandatory sodium regulation should inspire other African countries.

**Disclosures:** Dr Noubiap is supported by a Postgraduate Scholarship from the University of Adelaide.

## References

[1] GBD 2019 Diseases and Injuries Collaborators (2020). Global burden of 369 diseases and injuries in 204 countries and territories, 1990-2019: a systematic analysis for the Global Burden of Disease Study 2019. Lancet.

[2] GBD 2019 Risk Factors Collaborators (2020). Global burden of 87 risk factors in 204 countries and territories, 1990-2019: a systematic analysis for the Global Burden of Disease Study 2019. Lancet.

[3] World Health Organization (WHO) (2012). Guideline: Sodium Intake for Adults and Children.

[4] Mozaffarian D, Fahimi S, Singh GM, Micha R, Khatibzadeh S, Engell RE (2014). Global Sodium Consumption and Death from Cardiovascular Causes. N Engl J Med.

[5] Sixty-Sixth World Health Assembly (2013). Follow-Up to the Political Declaration of the High-Level Meeting of the General Assembly on the Prevention and Control of Non-Communicable Diseases. World Health Assembly.

[6] Trieu K, Neal B, Hawkes C, Dunford E, Campbell N, Rodriguez-Fernandez R (2015). Salt Reduction Initiatives around the World-A Systematic Review of Progress towards the Global Target. PLoS One.

[7] Webster JL, Dunford EK, Hawkes C, Neal BC (2011). Salt reduction initiatives around the world. J Hypertens.

[8] Bouhamida M, Benajibam N, Guennoun Y, Ait Lachguer S, Elhaloui NE, Zahrou F (2020). Implementing the national strategy of salt reduction in Morocco: The baker´s perspective. Pan Afr Med J.

[9] Joossens JV, Sasaki S, Kesteloot H (1994). Bread as a source of salt: an international comparison. J Am Coll Nutr.

[10] Charlton KE, Steyn K, Levitt NS, Zulu JV, Jonathan D, Veldman FJ (2005). Diet and blood pressure in South Africa: Intake of foods containing sodium, potassium, calcium, and magnesium in three ethnic groups. Nutrition.

[11] Mokhtar N, Elati J, Chabir R, Bour A, Elkari K, Schlossman NP (2001). Diet culture and obesity in northern Africa. J Nutr.

[12] Ouali MA, Derouiche A, Sqalli Houssaini T (2016). Le pain boulanger et sa teneur en sel. Néphrologie & Thérapeutique.

[13] Nwanguma BC, Okorie CH (2013). Salt (sodium chloride) content of retail samples of Nigerian white bread: implications for the daily salt intake of normotensive and hypertensive adults. J Hum Nutr Diet.

[14] Silva V, Padrão P, Novela C, Damasceno A, Pinho O, Moreira P (2015). Sodium content of bread from bakeries and traditional markets in Maputo, Mozambique. Public Health Nutr.

[15] Department of Health (2013). SA Regulations Relating to the Reduction of Sodium in Certain Foodstuffs and Related Matters. (Proclamation No. R. 214, 2013) Government Gazette, March 20 (Regulation Gazette No. 36274).

[16] Hofman KJ, Lee R (2013). Intersectorial Case Study: Successful Sodium Regulation in South Africa.

[17] Peters SAE, Dunford E, Ware LJ, Harris T, Walker A, Wicks M (2017). The Sodium Content of Processed Foods in South Africa during the Introduction of Mandatory Sodium Limits. Nutrients.

[18] Bertram MY, Steyn K, Wentzel-Viljoen E, Tollman S, Hofman KJ (2012). Reducing the sodium content of high-salt foods: effect on cardiovascular disease in South Africa. S Afr Med J.

[19] Noubiap JJ, Essouma M, Bigna JJ, Jingi AM, Aminde LN, Nansseu JR (2017). Prevalence of elevated blood pressure in children and adolescents in Africa: a systematic review and meta-analysis. Lancet Public Health.

[20] Nansseu JR, Noubiap JJ, Mengnjo MK, Aminde LN, Essouma M, Jingi AM (2016). The highly neglected burden of resistant hypertension in Africa: a systematic review and meta-analysis. BMJ Open.

[21] Noubiap JJ, Nansseu JR, Nkeck JR, Nyaga UF, Bigna JJ (2018). Prevalence of white coat and masked hypertension in Africa: A systematic review and meta-analysis. J Clin Hypertens (Greenwich).

[22] Luft FC, Miller JZ, Grim CE, Fineberg NS, Christian JC, Daugherty SA (1991). Salt sensitivity and resistance of blood pressure. Age and race as factors in physiological responses. Hypertension.

[23] Noubiap JJ, Bigna JJ, Nansseu JR (2015). Low sodium and high potassium intake for cardiovascular prevention: evidence revisited with emphasis on challenges in sub-Saharan Africa. J Clin Hypertens (Greenwich).

